# Dielectric Properties and Switching Processes of Barium Titanate–Barium Zirconate Ferroelectric Superlattices

**DOI:** 10.3390/ma11081436

**Published:** 2018-08-14

**Authors:** Alexander Sidorkin, Lolita Nesterenko, Yaovi Gagou, Pierre Saint-Gregoire, Eugeniy Vorotnikov, Nadezhda Popravko

**Affiliations:** 1 Physical Department, Voronezh State University, University sq. 1, 394018 Voronezh, Russia; lolita122@mail.ru (L.N.); vorotnikov.e@inbox.ru (E.V.); n-popravko@yandex.ru (N.P.); 2 Laboratoire de Physique de la Matière Condensée, Universite de Picardie Jules Verne, 80039 Amiens CEDEX, France; yaovi.gagou@u-picardie.fr; 3 UFR Sciences and Techniques, University of Toulon, 83041 Toulon CEDEX, France; pstgregoire@gmail.com; 4 Sciences and Arts, University of Nimes, 30021 Nimes CEDEX, France

**Keywords:** ferroelectric nanocomposites, smart materials, phase transitions, dielectric properties

## Abstract

This article is devoted to the investigation of the dielectric and repolarization properties of barium zirconate and barium titanate BaZrO_3_/BaTiO_3_ superlattices with a period of 13.322 nm on a monocrystal magnesium oxide (MgO) substrate. Synthesized superlattices demonstrated a ferroelectric phase transition at a temperature of approximately 393 °C, which is far higher than the Curie temperature of BaTiO_3_ thin films and bulk samples. The dielectric permittivity of the superlattice reached more than 10^4^ at maximum. As the electric field frequency increased, the dielectric constant of the studied superlattice decreased over the entire study temperature range, but position of the maximum dielectric constant remained the same with changing frequency. The temperature dependence of the inverse dielectric permittivity 1/ε(T) for the studied samples shows that, in the investigated superlattice, both Curie–Weiss law and the law of “two” were followed. Additionally, the ε(T) dependences showed practically no temperature hysteresis with heating and cooling. Samples of synthesized superlattices had a relatively small internal bias field, which was directed from the superlattice towards the substrate.

## 1. Introduction

Recently, in the physics of solid state nanostructures and in physical materials science, much attention has been paid to artificially created layered formations—ferroelectric superlattices. These are periodic multilayer epitaxial structures consisting of consecutively applied layers of different ferroelectric or ferroelectric and dielectric materials. Both fundamental and applied interests in these structures are primarily related to their many physical parameters (e.g., Curie temperature, spontaneous polarization, dielectric constant, and switching peculiarities), which differ significantly in comparison with one-component thin films or bulk parent materials [1,2,3,4]. It is essential that these parameters can be changed in the right direction via the appropriate structural variation of the superlattice.

In practice, when creating ferroelectric superlattices, most attention is paid to various combinations of perovskite group materials as initial components. This is firstly because of the relative simplicity of their structure, and because the properties of these compositions have been studied intensively in the literature. The most frequently considered structures are bilayer superlattices, where one of the recurring layers is ferroelectric barium titanate (BaTiO_3_) and the second is either a different ferroelectric (e.g., lead titanate, PbTiO_3_ (BT/PT lattice) [5]), or a dielectric (e.g., strontium titanate, SrTiO_3_ (BT/ST lattice) [6,7,8]), a paraelectric (e.g., barium zirconate, BaZrO_3_ (BT/BZ lattice) [9,10,11,12,13,14,15]), or a relaxor (e.g., barium zirconate titanate, BaTi_0.62_Zr_0.32_O_3_ (BT/BZT lattice) [16]). 

Structural studies using X-ray diffraction and Raman scattering methods are predominant among the experimental studies of these formations [12,14,16,17,18]. Dielectric and repolarization studies are most commonly used to describe the properties of materials that are suggested for practical applications [19,20,21,22].

The theoretical description of the properties of ferroelectric superlattices was done from first principles within density functional theory [23,24,25], and calculations within the framework of the Landau–Ginsburg theory [26,27,28,29,30]. The main achievement of the first principles calculations is the ability to predict the structure of the ground state and the direction of polarization in the considered ferroelectric superlattices. The great success of the Landau–Ginzburg phenomenological theory in describing the peculiarities of phase transformations and numerous properties of bulk ferroelectrics served as the basis of its use to describe the size effects in ferroelectric films, and then in superlattices, by introducing appropriate boundary conditions on the boundary with the substrate and special link parameters for the adjacent layers [31]. The dynamics of the switching processes in ferroelectric superlattices is described mainly in the framework of the Landau–Khalatnikov theory [32,33].

The main factor determining the properties of these structures is considered to be mechanical stresses arising from the differences between the sizes of elementary cells of the components and the substrate. Another factor is the appearance of charges in the area where layers with different polarization contact each other, along with a change in the chemical composition and chemical bonds in the near-surface layers, etc. [34,35,36,37,38]. The mentioned changes occur even in epitaxial layer-by-layer superlattice growth with perfectly flat layer surface contact areas. If the newly applied layer grows on the damaged surface, different structural defects appear in the contact area. As a result of changes in the structure, and the nature of interactions between structural units in the interlayer contacts, the properties of the synthesized multilayer objects and the features of the phase transitions in the superlattices are also changed in comparison with the pattern bulk or film materials. 

A new direction of modern material science called epitaxial engineering has emerged on the basis of these fundamental effects. It aims at the development and implementation of multilayer ferroelectric superlattices created by the consistent confluence of separate layers of various ferroelectric or ferroelectric and dielectric materials [9]. Deformation engineering is a more formally narrow, but extremely productive part of this concept, that considers and employs epitaxial stresses arising in the contact area of different layers as the leading factor determining the properties of superlattices and thin ferroelectric films on a substrate.

Experience shows that the structure and properties of ferroelectric superlattices essentially depend on their period *Λ*, which is the total thickness of periodically repeating various layers of the superlattice. According to the logic of conjugation conditions (primarily, the mismatch stresses and the experimental data), the greatest changes in the properties of superlattices take place in the region of finite values of period *Λ*, limited both from the small and from the relatively large values [12].

The interlayer stresses caused by mismatch deformations of the structure of superlattice layers increase as the thickness of the conjugated layers decreases. Conversely, these stresses decrease as the period of the superlattice increases, because of the relaxation of interlayer stresses through the formation of misfit dislocations. In this case, in the limit of large periods, the synthesized lattice behaves as a sum of noninteracting layers.

For very small periods of the superlattice (two elementary cells in each of the layers composing the lattice), the set of conjugated layers constitutes a solid solution of components of the superlattice. The value of the polarization in this structure is less than for the superlattice. In addition, interlayer diffusion occurs at very short superlattice periods, and as in the case of mismatch dislocations, it reduces the stresses generated by the lattice discrepancies between its layers.

As a result, the polarization of the superlattice and the properties it causes generally have increased values in the limited interval of period values. For example, for BT/BZ superlattice, this interval is 16 Å ≤ *Λ* ≤ 500 Å, and the best properties of superlattices correspond to the median values of the indicated period *Λ* [12].

Although we have an understanding of the mechanisms which control the properties of the superlattice in comparison with a homogeneous material, the existing information on the various properties of ferroelectric superlattices is clearly insufficient. In this regard, the present paper is an attempt to expand the completeness of description of the properties of ferroelectric superlattices in the example of the BT/BZ superlattices. These superlattices were chosen because of their expected giant dielectric constant values, and also their potential for application in non-volatile ferroelectric memory devices.

## 2. Materials and Methods 

The present studies were carried out on perovskite-like ferroelectric superlattices consisting of 32 BaZrO_3_/BaTiO_3_ alternating layers with a thickness of 6.65 nm for barium zirconate layers and 6.67 nm for barium titanate layers, with a superlattice period of *Λ* = 13.32 nm that was layered by pulsed laser sputtering. The BT and BZ epitaxial layers were deposited on a substrate of monocrystalline magnesium oxide MgO with (001) orientation (*a_MgO_* = 4.213 Å), with a sublayer of La_1/2_Sr_1/2_CoO_3_ conducting oxide (LSCO) (*a_LSCO_* = 3.805 Å) as a lower electrode, and a platinum top electrode with a diameter of 1 mm.

The thickness of the continuous lower LSCO electrode was 50 Å. The electrode was deposited on the substrate at a temperature of 750 °C with a partial oxygen pressure of 0.2 mbar. The temperature and oxygen pressure during application of BT and BZ layers were, respectively, 750 °C and 0.1 mbar. The deposition rate of these layers was 0.42 Å per pulse and 0.35 Å per pulse for BT and BZ layers, respectively. The above-mentioned thicknesses of the individual layers of barium zirconate and barium titanate were calculated by multiplying the deposition rate by the number of pulses. The deposition chamber was equipped with a 15 kV reflection high-energy electron diffraction (RHEED) system. With its help, the surface quality of the layers composing superlattices was systematically monitored at various growth steps. The RHEED bands obtained from the BZ and BT layers and their azimuth positions showed the ideal epitaxial growth of the cube on the cube. Sample deposition details and an example of the corresponding RHEED picture are given in [14]. 

X-ray diffraction studies were carried out using cobalt anode as a source of X-radiation with wavelengths *λ_α_*_1_ = 1.7889 Å, *λ_α_*_2_ = 1.7928 Å (not used), *λ_β_* = 1.6208 Å.

The temperature dependence of the dielectric permittivity of test samples was found by measuring the sample capacity with the Instek LCR-819 LCR-meter. The frequency of the measurement signal was in the range of 1–50 kHz and the measuring voltage was 0.1 V. In addition, the dependences of the coercive field and spontaneous polarization on temperature, including the Curie temperature, were studied by dielectric hysteresis loops, using the modified Sawyer–Tower compensation scheme.

## 3. Results

**Figure 1:**
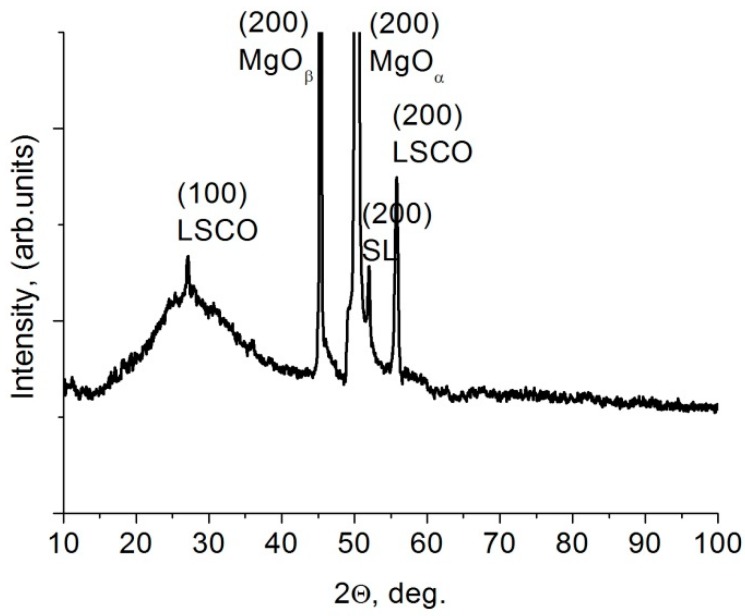
Diffractogram of barium zirconate–barium titanate superlattice.

The X-ray diffraction studies demonstrated that the investigated BaZrO_3_/BaTiO_3_ superlattices represented perfect monocrystalline structures, as evidenced by one corresponding reflex, marked as SL in Figure 1. Its interplanar distance was *a* = 4.084 Å, which does not correspond to either the barium titanate or the barium zirconate compound. The diffraction pattern also showed the two most intense *α* and *β* lines from the MgO (200) substrate with *a* = 4.212 Å and two reflexes from the lower LSCO electrode with *a* = 3.826 Å.

It is known that in a free state at room temperature, barium zirconate is a paraelectric without polarization. The sizes of the elementary cells of barium titanate and barium zirconate along the substrate were different. The bulk BT at room temperature had a tetragonal structure with dimensions *a_BT_* = 3.992 Å and *c_BT_* = 4.036 Å, and the adjacent BZ layer for the bulk material had a cubic structure with the size *a_BZ_* = 4.192 Å. Due to the indicated difference in the size of elementary cells under the action of the mismatched strain stresses in the superlattice, the unit cell BZ was compressed from the direction parallel to the substrate. As a result, in the BZ layers, a polarization was induced in the direction perpendicular to the substrate and the lattice BaZrO_3_/BaTiO_3_ as a whole, in accordance with research of the dielectric hysteresis loops [12], demonstrating the reversible polarization in the direction normal to the substrate.

With the aim of revealing the regularities of the influence of the layer material and the dimensional parameters of the layers of the ferroelectric superlattice on its dielectric characteristics and phase transformations, in the present work, temperature studies of the dielectric constant and the tangent of the dielectric loss angle of the indicated ferroelectric superlattices were carried out.

**Figure 2:**
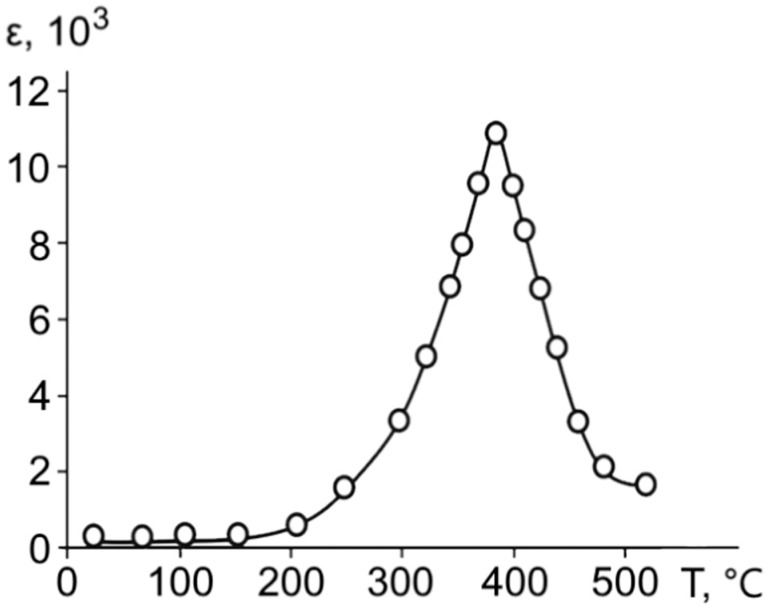
The temperature dependence of dielectric permittivity of the ferroelectric BaZrO_3_/BaTiO_3_ superlattice under a measuring field with a frequency of 1 kHz.

Studies of the temperature dependence of the dielectric constant showed the presence of a single maximum corresponding to a phase transition in the superlattice with a temperature of about 393 °C (Figure 2), which is substantially higher than the transition temperature for bulk and film samples of barium titanate. The dielectric constant of the synthesized superlattice increased from about 10–50 at room temperature to approximately 11,000 units at the Curie point. This value of *ε* is higher than that for many known ferroelectric films. The tangent of the dielectric loss angle in the polar phase retained values in the order of 5 × 10^−3^ in the entire investigated temperature range, dropping by about an order of magnitude upon transition in the nonpolar phase.

**Figure 3:**
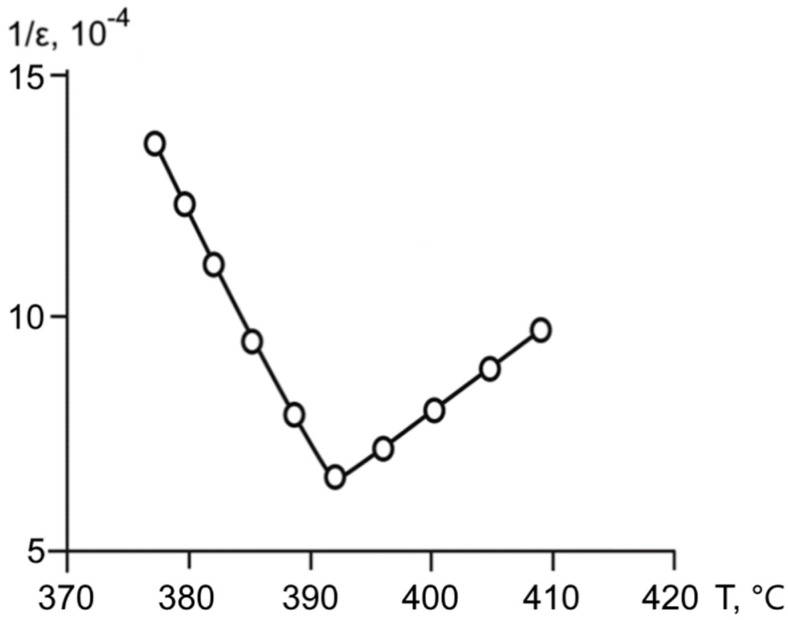
Dependence of the reverse dielectric permittivity on the temperature for BaZrO_3_/BaTiO_3_ superlattice.

The temperature dependence of the inverse dielectric permittivity 1/*ε*(*T*) for the studied samples (Figure 3) shows that, in the investigated superlattice, both Curie–Weiss law and the law of “two“ were followed. The permittivity near the Curie point was inversely proportional to the temperature, and the ratio of the slopes of the inverse dielectric constant below and above the Curie point was close to 2 (in reality, it was equal to 1.97:1.83; Figure 3). Additionally, the *ε*(*T*) dependences showed practically no temperature hysteresis with heating and cooling.

**Figure 4:**
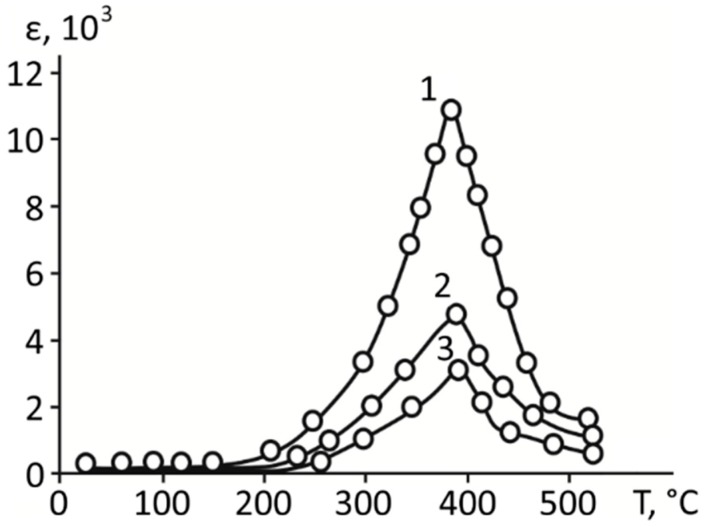
The dependence of dielectric permittivity on temperature for BaZrO_3_/BaTiO_3_ superlattices with different frequency of measuring field: Lines 1, 2, and 3 correspond to 1 kHz, 10 kHz, and 50 kHz, respectively.

The results of studying the effect of frequency on the values of dielectric response are shown in Figure 4. From these dependencies, it is seen that the dielectric constant decreased with increasing measuring field frequency throughout the investigated temperature range. At the same time, the position of the temperature dependence maximum of permittivity remained relatively stable with changing frequency.

This behavior evidently indicates the presence of areas in the studied materials with different relaxation times, which respond to an applied action with different rates, giving (or not giving) their contribution to the measured dielectric response.

**Figure 5:**
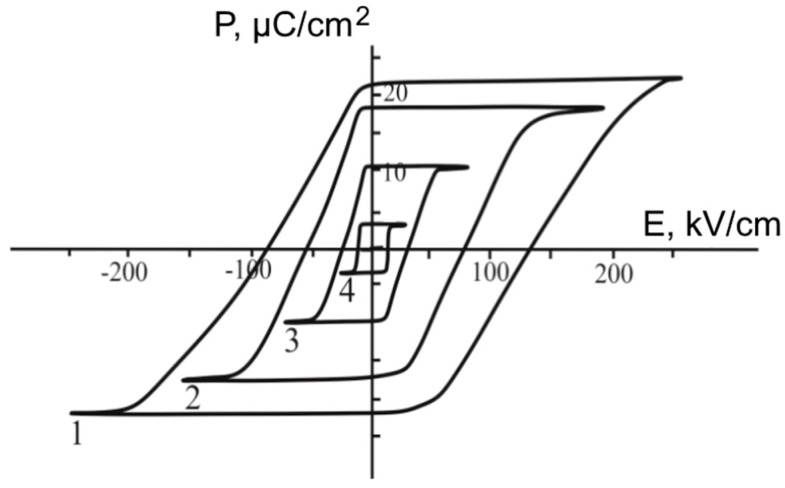
Dielectric hysteresis loops for BaZrO_3_/BaTiO_3_ superlattices at the different temperatures: Curves 1, 2, 3, and 4 correspond to 20 °С, 340 °С, 380 °С, and 395 °С, respectively.

Active attention to the switching process of ferroelectric thin-film structures and superlattices is due to the possibility of achieving the necessary switching characteristics of such materials for their use in ferroelectric memory devices. A classical way of determining the key parameters characterizing the possibility of using the studied material in switching devices is the study of dielectric hysteresis loops using the Sawyer–Tower scheme. Figure 5 demonstrates dielectric hysteresis loops for the studied superlattices at different temperatures. It is seen that this material is characterized by rectangular dielectric hysteresis loops, creating good opportunities for fixing a required value of polarization by electric fields from a certain range.

**Figure 6:**
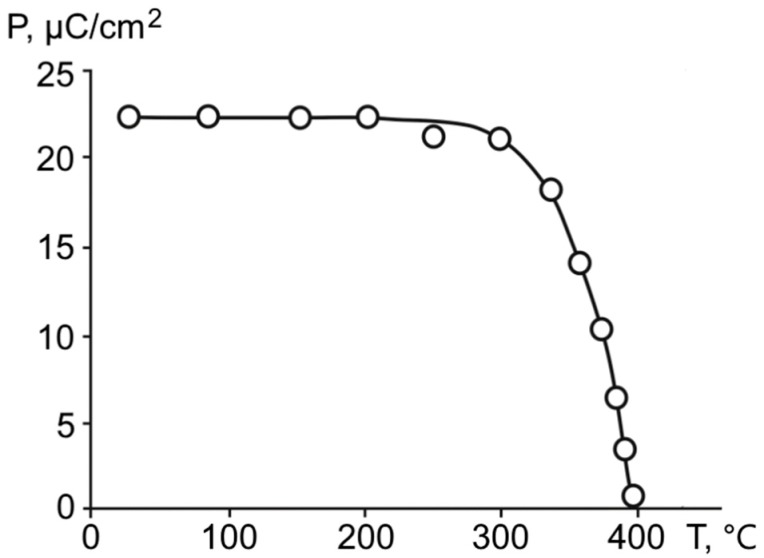
The temperature dependence of the spontaneous polarization for the ferroelectric BaZrO_3_/BaTiO_3_ superlattice.

**Figure 7:**
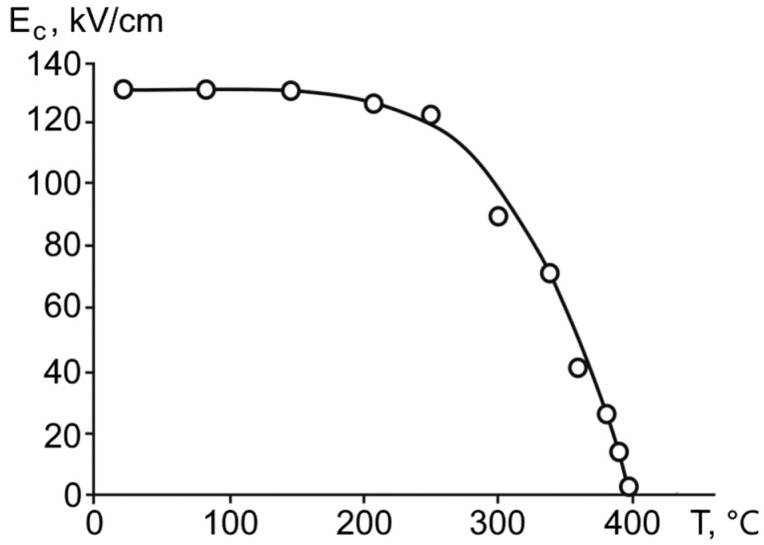
The temperature dependence of the coercive field for the ferroelectric BaZrO_3_/BaTiO_3_ superlattice.

According to these dielectric hysteresis loops, the temperature dependencies of the spontaneous polarization (Figure 6) and the coercive field (Figure 7) were calculated. The calculations showed that the created structures are characterized by a sufficiently high value of spontaneous polarization: about of 22.0 μС/cm^2^ at room temperature. From Figure 6, it is clearly visible that superlattices are characterized by a smoother decrease in the polarization as they approached the temperature of the transition to a nonpolar phase, compared with the mother barium titanate bulk material, where the polarization behavior demonstrated a sharp decrease near the phase transition temperature.

The coercive field of the superlattice was equal to *E_c_* = 130 kV/cm at room temperature (20 °С). The temperature dependence of the coercive field occupies a rather wide range of temperatures, similar to the behavior of decreasing spontaneous polarization (Figure 7).

The total thickness of the synthesized lattice was about 400 nm. Multiplying the obtained value of the coercive field *E_c_* = 130 kV/cm by the thickness of the superlattice, it is easy to see that this field (and hence the switching of the superlattice) could be achieved by applying a voltage of the order of 5 V-typical for modern low-voltage control electronic devices. This allows ferroelectric superlattices to be integrated into standard silicon chips [39]. This is an additional argument for the use of ferroelectric superlattices in the form of integrated ferroelectrics in many nanodevices, a vivid example of which is the development of ferroelectric memory elements, where a logical unit corresponds to a ferroelectric domain of a certain direction [40,41].

**Figure 8:**
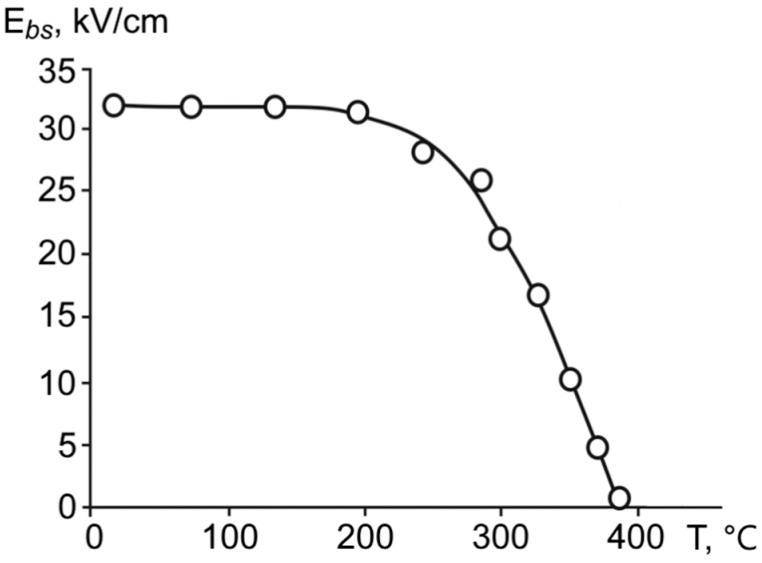
The temperature dependence of internal bias field for ferroelectric BaZrO_3_/BaTiO_3_ superlattices.

Studies have shown that samples of synthesized superlattices have an internal bias field, which was determined from the displacement of the dielectric hysteresis loop along the field axis. According to the calculations, the internal field in the studied superlattice was equal to 32 kV/cm at room temperature (20 °С), and it slightly reduced as the temperature increased (Figure 8).

To form an internal field, the ferroelectric structure must be asymmetric with respect to the polar direction. In real materials, there are traditionally three reasons for this: (1) the presence of an ordered system of polar defects in the sample; (2) differences in the materials of the electrodes on opposite surfaces of the ferroelectric plate; and (3) the presence of a substrate [42]. In our structures, there are no ordered polar defects, and so the last two reasons remain. Differences in the materials of the electrodes with different work function of the electrons lead to the formation of electron clouds with different concentrations on the different surfaces of the ferroelectric, and hence, lead to the formation of an internal field. In our case, the lower electrode is chemically complex, so it is difficult to evaluate the role of this cause. The third reason certainly had an effect in our case, since the materials of both the lower electrode and the substrate had different unit cell sizes compared to the materials of the superlattice. Therefore, there were mismatch stresses near the contacting surface of the superlattice, causing the appearance of an internal field. To test the reality of the role of this mechanism in the creation of an internal field in our case, it is necessary to determine the sign of the internal field acting in the superlattice under study.

**Figure 9:**
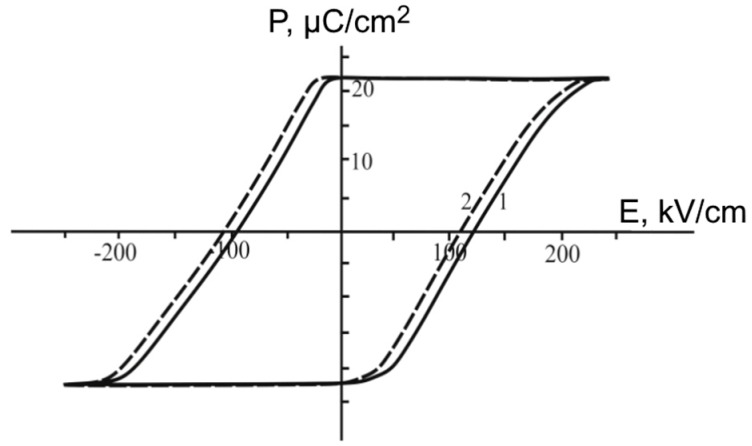
Dielectric hysteresis loop for BaZrO_3_/BaTiO_3_ superlattice at the temperature of 20 °С: 1—without internal bias field, 2—with additionally applied field.

A special study was carried out to determine the sign (direction) of the indicated field. A constant field of predetermined direction was applied to the superlattice and shifting of the hysteresis loop was registered in the total switching and additionally applied constant field (Figure 9). 

As can be seen from the given figure, when a positive charge was applied to the substrate, the hysteresis loop shifted to the side opposite to its displacement only under the action of the internal bias field in the sample. This means that the initial internal bias field was directed from the surface of the superlattice to the substrate.

## 4. Discussion and Conclusions

Summarizing the results of our research, the following can be noted. The present studies were carried out on superlattices consisting of 32 layers of barium titanate and barium zirconate with period *Λ* = 13.32 nm, which were applied to a magnesium oxide substrate by pulsed laser deposition. Diffractometric studies showed that the studied samples had perfect single-crystal structures.

These superlattices had a ferroelectric phase transition at temperatures approximately equal to about 393 °С, which is significantly higher than the Curie point in bulk samples and thin films of barium titanate. Implementation in a fairly wide range of temperatures of the “two” law, the absence of temperature hysteresis of the dielectric constant, smooth changes in the polarization, and coercive field with a temperature near the Curie point indicated that the observed phase transition in the superlattices exhibited characteristic features of a second-order phase transition. However, it was observed in the experiment that the Curie temperatures (T_C_) and Curie–Weiss temperature (T_0_) did not coincide. Consequently, this question requires additional consideration.

As in ferroelectric composites, the values of the dielectric constant in the superlattices decreased with increasing measuring field frequency in the whole studied temperature range. At the same time, the position of maximum of the dielectric permittivity remains constant as the frequency changed.

It is reasonable to consider that polarization in the barium zirconate layer in the synthesized superlattice was induced by the mechanical stresses caused by the adjacent layers of barium titanate. The general increase of the Curie point in the superlattice in comparison to barium titanate in the bulk or film samples may be related to the mutual elastic action of the barium titanate and barium zirconate layers on each other. These stresses apparently changed the general structure of the thermodynamic potential of the superlattice through the destruction of the simultaneous existence of the local minima with zero and nonzero polarization in all temperature ranges, which is characteristic of a first-order phase transition. Thus, the type of phase transition can change.

To explain the direction of the internal biasing field obtained in the researched structures, one can use the representations of the flexoelectric effect. The size of a unit cell of the lower electrode (which is located near the substrate) is less than the unit cell size of the barium zirconate or barium titanate material in contact with it. This creates compressive stresses acting on the unit cell of the superlattice material in contact with it in the direction parallel to the substrate plane. As a result of these stresses, the elementary cells of the superlattice material contacting this electrode take the shape of a trapezoid, and thus, its short base contacts the electrode. Due to the change in the shape of the unit cell, the positively charged titanium or zirconium ion located in its center is extruded to the side opposite to the substrate, which means the appearance of the opposite negative charge at the interface with the electrode.

The values of the coercive fields that we found indicate that the superlattices under consideration could be switched by voltages of about 5 V, which is characteristic for controllers of low-voltage semiconductor devices, which is an additional argument for their use in modern nanodevices [39].
